# Methodology for the development of National Multidisciplinary Management Recommendations using a multi-stage meta-consensus initiative

**DOI:** 10.1186/s12874-022-01667-w

**Published:** 2022-07-11

**Authors:** John C. Hardman, Kevin Harrington, Tom Roques, Sanjai Sood, Jemy Jose, Shane Lester, Paul Pracy, Ricard Simo, Costa Repanos, Frank Stafford, Chris Jennings, Stuart C. Winter, Hugh Wheatly, Jarrod Homer, B. Nirmal Kumar, Vinidh Paleri

**Affiliations:** 1 Head and Neck Unit, The Royal Marsden NHS Foundation Trust, London, SW3 6JJ UK; 2 Department of Oncology, Norfolk and Norwich University Hospitals NHS Foundation Trust, Norwich, UK; 3 Department of Otolaryngology, Head and Neck Surgery, Bradford Teaching Hospitals NHS Foundation Trust, Bradford, UK; 4 Department of Otolaryngology, Head and Neck Surgery, Hull and East Yorkshire Hospitals NHS Trust, Hull, UK; 5 Department of Otolaryngology, Head and Neck Surgery, South Tees Hospitals NHS Foundation Trust, Middlesbrough, UK; 6 Department of Otolaryngology, Head and Neck Surgery, University Hospitals Birmingham NHS Foundation Trust, Birmingham, UK; 7 Department of Otolaryngology, Head and Neck Surgery, Guy’s and St Thomas’ NHS Foundation Trust, London, UK; 8 Department of Otolaryngology, Head and Neck Surgery, Portsmouth Hospitals NHS Trust, Portsmouth, UK; 9 Department of Otolaryngology, Head and Neck Surgery, South Tyneside and Sunderland NHS Foundation Trust, Sunderland, UK; 10 Department of Otolaryngology, Head and Neck Surgery, Oxford University Hospitals NHS Foundation Trust, Oxford, UK; 11 Department of Otolaryngology, Head and Neck Surgery, Gloucestershire Hospitals NHS Foundation Trust, Gloucester, UK; 12 Department of Otolaryngology, Head and Neck Surgery, Manchester University NHS Foundation Trust, Manchester, UK; 13 Department of Otolaryngology, Head and Neck Surgery, Wrightington Wigan & Leigh NHS Foundation Trust, Wigan, UK

**Keywords:** Delphi, Audit, Cost effectiveness, Guidelines, Head and neck cancer, Unknown primary

## Abstract

**Background:**

Methods for developing national recommendations vary widely. The successful adoption of new guidance into routine practice is dependent on buy-in from the clinicians delivering day-to-day patient care and must be considerate of existing resource constraints, as well as being aspirational in its scope. This initiative aimed to produce guidelines for the management of head and neck squamous cell carcinoma of unknown primary (HNSCCUP) using a novel methodology to maximise the likelihood of national adoption.

**Methods:**

A voluntary steering committee oversaw 3 phases of development: 1) clarification of topic areas, data collection and assimilation, including systematic reviews and a National Audit of Practice; 2) a National Consensus Day, presenting data from the above to generate candidate consensus statements for indicative voting by attendees; and 3) a National Delphi Exercise seeking agreement on the candidate consensus statements, including representatives from all 58 UK Head and Neck Multidisciplinary Teams (MDT). Methodology was published online in advance of the Consensus Day and Delphi exercise.

**Results:**

Four topic areas were identified to frame guideline development. The National Consensus Day was attended by 227 participants (54 in-person and 173 virtual). Results from 7 new systematic reviews were presented, alongside 7 expert stakeholder presentations and interim data from the National Audit and from relevant ongoing Clinical Trials. This resulted in the generation of 35 statements for indicative voting by attendees which, following steering committee ratification, led to 30 statements entering the National Delphi exercise.

After 3 rounds (with a further statement added after round 1), 27 statements had reached ‘strong agreement’ (*n* = 25, 2, 0 for each round, respectively), a single statement achieved ‘agreement’ only (round 3), and ‘no agreement’ could be reached for 3 statements (response rate 98% for each round). Subsequently, 28 statements were adopted into the National MDT Guidelines for HNSCCUP.

**Conclusions:**

The described methodology demonstrated an effective multi-phase strategy for the development of national practice recommendations. It may serve as a cost-effective model for future guideline development for controversial or rare conditions where there is a paucity of available evidence or where there is significant variability in management practices across a healthcare service.

**Supplementary Information:**

The online version contains supplementary material available at 10.1186/s12874-022-01667-w.

## Background

Various methods have been employed to generate national guideline recommendations [1–3]. The management of head and neck squamous cell carcinoma of unknown primary (HNSCCUP) has been the subject of a number of these methods which have been used to produce the most widely referenced recommendations [4–9]. However, the successful adoption of new guidance into standard practice is not just dependent on assimilation of the best available evidence, but also relies on buy-in from the clinicians delivering day-to-day patient care. Guidelines must be considerate of existing resource constraints, as well as being aspirational in their scope. Current strategies to generate guidelines may involve meetings behind ‘closed doors’ and/or rely on a handful of ‘experts’ not familiar with practice preferences and limitations across the healthcare system [2, 5].

The Delphi process is often used in scenarios where refining a group opinion is desired and has previously been successfully implemented in head and neck oncology [10–12]. It relies on anonymised responses, to reduce bias from dominant individuals, and iterative feedback, to provide group pressure towards conformity [13]. A Delphi exercise may be used to allow a wider gamut of stakeholders to review a set of draft recommendations before adoption, even if the recommendations have been generated using robust procedures and based on best available evidence with little equipoise. This may have two benefits: firstly, it allows for a greater number of individuals to input into the process than may be able to attend a time-constrained event such as a consensus day, and secondly, it may help to consolidate more universal buy-in to the resultant output as more stakeholders have been included in production.

HNSCCUP is a condition where the patient presents with metastatic cancer in the lymph nodes of the neck but where the original site of the cancer (which is usually thought to be from the mucosa lining the upper aerodigestive tract) cannot be identified on clinical examination, imaging investigations or diagnostic biopsies.4 It accounts for roughly 3-5% of head and neck cancers, which itself is the 8th most common form of cancer in the UK [14–16].

Multidisciplinary team (MDT) meetings in healthcare are already set up to consider relevant evidence and deliver consensus opinions. They are mindful of patient wishes and requirements, and of their own local practice capabilities [17]. As such, they are well placed to consider the impact of clinical recommendations on the healthcare they can deliver. Whilst individual MDTs may have particular biases towards more dominant specialties therein, an exercise that seeks to include all MDTs in a given field would be able to mitigate much of this local variation to maximise the suitability of any consensus guidelines agreed upon.

Additionally, funding is not always available to support the development of clinical practice guidelines. This may be a particular issue for rarer conditions that may struggle to attract similar funding to more common diseases that can attract more attention. Consequently, methodologies that can utilise voluntary effort and can delegate the workload to multiple stakeholders, may be desirable.

### Objective

This initiative aimed to produce National Guidelines for the management of head and neck squamous cell carcinoma of unknown primary (HNSCCUP) using a novel multi-phase meta-consensus methodology.

## Methods

The ENT UK Head and Neck Society Council and a Clinical Research Fellow formed the Steering Committee to oversee the initiative (first-line authors). The Clinical Research Fellow and Doctoral Supervisor (also a Steering Committee Member) adopted central leadership roles to maintain project momentum. The development of recommendations was divided into 3 phases: 1) clarification of topics and data assimilation; 2) a National Consensus Day; and 3) a National Delphi Exercise. An outline of the methodology was published online and shared with participants in advance of phase 2, at https://bit.ly/HNSCCUPconsensusprocess.

### Phase 1: clarification of topics and data assimilation

#### Identification of topics to be investigated through systematic reviews

Topics felt to be amenable to systematic review of the published literature were selected and the specific research question agreed by the steering committee (Supplementary material). Consultants who were identified as national experts in their specialty, with appropriate experience of critical appraisal, were approached to supervise senior trainees and clinical fellows delivering these reviews. The systematic review teams were encouraged to engage with their local library and information specialist to facilitate the development of suitable and effective search strategies. The agreed minimum output was a presentation of the results during the National Consensus Day, though write-up for publication was also encouraged.

#### Identification of topics to be presented by expert stakeholders

For topics not felt amenable to systematic review, expert stakeholders were approached to assimilate the literature with an agreed output of a presentation for the National Consensus Day.

#### Identification of data to be collated from National Audit of practice

To learn from the contemporary management of HNSCCUP patients in the UK, a National Audit was conducted in collaboration with INTEGRATE (The UK ENT Trainee Research Network). The full methodology is outlined in a separate publication. In brief, all UK centres managing HNSCCUP patients were invited to participate via mailouts from ENT UK, the Association of Otolaryngologists in Training (AOT) and the INTEGRATE network. Patients undergoing positron emission tomography combined with computed tomography (PET-CT) for the identification of a primary site cancer, having presented with cervical metastases without a clinically evident primary site between 2015 and 2020, were eligible for inclusion. Pathway data were collected to understand the patient’s diagnostic journey and outcome data were collated with a median follow up of 30 months for survivors. Methodology was agreed by the HNSCCUP Consensus Steering Committee.

#### Interim reports from ongoing clinical trials

The Chief Investigators of ongoing clinical trials relevant to the management of HNSCCUP were approached to outline the research design and outputs, and to see if they were able to present any interim results relevant to the recommendations being considered.

### Phase 2: National Multidisciplinary Consensus event to generate draft statements

#### Draft statements generated by section chairs in advance of event

In advance of the Consensus Day, all presentations of evidence outlined in phase 1 were shared with chairs for each of four sessions, focused around key steps in the management pathway: 1) investigations for clinically suspected HNSCCUP; 2) diagnostic surgery to try and identify the primary site; 3) surgical treatments; and 4) non-surgical treatments. Chairs reviewed the evidence and generated draft consensus statements using NICE guidance for recommendation language.2 This included the use of the term ‘offer’ to reflect a strong recommendation, where there is felt to be clear evidence of benefit, and use of the term ‘consider’ for a recommendation where the evidence of benefit is felt to be less certain. The evidence and draft statements were subsequently shared with delegated breakout group leads who would be leading discussions on the consensus day, to incorporate any feedback prior to further dissemination/development.

#### Presentations of evidence to event attendees

The consensus day was a hybrid event, accepting both virtual and in-person attendees, structured around four sessions which reflected the patients’ diagnostic and treatment pathways (Supplementary material). All presentations were pre-recorded to facilitate the generation of draft consensus statements as above.

#### Breakout group discussions to amend statements

At the end of each of the four sessions, both virtual and in-person attendees were split into equal-sized breakout groups. Each breakout group was chaired by a pre-identified attendee who had advanced access to the evidence used to generate the draft statements ahead of the day. Individual breakout groups were allocated unique pre-drafted statements to discuss and revise as appropriate, including generating new statements or removing statements entirely. If time allowed, groups were able to discuss statements allocated to other groups too.

Statements were edited by the breakout group lead live on an online Google Document. Once the breakout groups were brought back together, the session chair invited the group leads to summarise their discussions and any revisions made to the statements. This was then opened up to all attendees for input. Edits were again made live on the Google Document while discussions proceeded.

#### Indicative voting on draft statements

At the end of each session, the draft consensus statements were transferred to an online voting system (sli.do) which was accessible via a weblink and/or QR code. Attendees were invited to indicate their support (agree/disagree) for each statement. Voting remained open for a minimum of 90 minutes. The raw results of the indicative vote were disseminated to attendees alongside feedback requests the day after the meeting.

### Phase 3: Delphi exercise leading to national adoption of recommendations

#### Ratification of draft statements for clarity and consistency of wording by steering committee

The steering committee reviewed all draft statements from the consensus day to ensure consistency of style, and phrasing. Finalised statements for the Delphi process were piloted amongst the steering committee for readability and suitability of the Google Forms platform.

#### Three rounds of online Delphi voting with consensus view from all national MDTs

Following ratification by the Steering Committee, representatives from each UK HN MDT were invited to participate in an online modified Delphi process, hosted on Google Forms, recording their support for each statement with a binary agree/disagree response.

##### Schedule

 The following schedule was employed:

- 10 days for MDT responses, to ensure time for discussion at a weekly MDT meeting.
- 4 days for chasing final responses, analysis and preparation of statements for the next round.

##### Thresholds

Up to three rounds of the Delphi process were planned, with thresholds as follows:

- ≥80% strong agreement (≤20% strong disagreement)
- ≥67% agreement (≤33% disagreement) (applied only after 3rd round)

##### Achieving consensus 

The following strategies to achieve consensus were set out a priori:

- Statements reaching the ‘strong agreement’ threshold at any stage will be removed from further rounds.
- After round 1, statements using the term ‘offer’ which do not achieve ‘strong agreement’ will be duplicated with the term ‘consider’ in place of ‘offer’ for subsequent rounds. Both the ‘offer’ and ‘consider’ statements will be presented in parallel for subsequent rounds.
- After round 3, if both the ‘offer’ and ‘consider’ statements achieve the same level of agreement, then the ‘offer’ statement will be adopted in preference.
- After round 3, if the ‘consider’ statement achieves ‘strong agreement’ and the ‘offer’ statement achieves ‘agreement’, then the ‘consider’ statement will be adopted.
- Action terms like ‘Perform/refer/include’ will be considered to have the same impact as ‘offer’ terms/statements as above.

Comments were invited at each round. The Steering Committee considered any feedback given by participants for incorporation into subsequent rounds. The Steering Committee was the final arbitrator of amendments between rounds and the ultimate production of the consensus statements.

Please note that statements drafted with ‘consider’ terminology at the Consensus Day in Phase 2 could not be ‘upgraded’ to ‘offer’ during the Delphi process in Phase 3. It was felt that the best opportunity to appraise the available evidence for certainty of clear benefit was at the Consensus Day and that if the higher threshold was not felt to have been met at this stage, then it should not be subsequently overridden.

#### Final ratification of adopted statements by stakeholder organisations

The finalised consensus statements were distributed to the representatives of all UK HN MDTs for endorsement. Accepted statements were incorporated into the 6th edition of the ‘United Kingdom National Multidisciplinary Guidelines for Head and Neck Cancer’. This document was endorsed by all UK HN MDT stakeholder organisations.

### Deviations from a priori methodology

Pre-recorded presentations were late to arrive from some speakers which limited the time that some session chairs and delegated breakout group leads had to generate the draft consensus statements ahead of the consensus event. As such, the intention to share draft statements with consensus day attendees was necessarily abandoned.

During the first session discussions on the Consensus Day, virtual participants were limited to having their comments/questions fielded through the written chat portal due to technical difficulties with the audio link.

Where ‘offer’ and ‘consider’ statements were presented in parallel, if the respondent indicated support for ‘offer’ but not ‘consider’ they were reminded that the statements would be analysed separately.

## Results

At the outset of the exercise, the Steering Committee used its national networks to compile a comprehensive list of 58 HN MDTs throughout the UK. Additionally, an ENT UK contact was identified who sat on each of these MDTs and who agreed to act as the MDT representative for the forthcoming Consensus Process.

### Consensus day

The National Consensus Day was attended by 227 participants (54 in-person and 173 virtual). Representatives at the were from ENT (70%), Clinical Oncology (18%), Radiology (4%), Plastic Surgery (2%), Histopathology (1%), Speech and Language Therapy (1%), Maxillofacial Surgery (1%), and other specialties (3%).Within the four sessions, there were 20 pre-recorded presentations delivered by 39 health professionals: 7 novel systematic reviews; 7 expert stakeholder viewpoints, 3 focused summaries of interim data from the National Audit; and 3 presentations from ongoing Clinical Trials (MOSES NCT04151134 and FIND NCT03281499). After each of the four sessions, attendees were divided into 5 breakout groups (2 in-person, 3 virtual) to scrutinise the statements that had been pre-drafted, having been presented with the best available evidence.

Following subsequent discussions amongst all attendees (virtual and in-person) led by the session chairs, 29 statements were agreed upon for indicative voting. The response rate for each statement varied between 61 and 115 indicative votes (median *n* = 91), with agreement ranging between 62.3 and 98.1% (median 90.2%) (Supplementary material).

The Consensus Day received income from ticket sales (both virtual and in-person) and from exhibitor fees. Costs were related to venue hire, catering, information technology resources (the Zoom online platform and sli.do voting subscription) and event coordinator time. There was a net profit from the day which was distributed to ENT UK and the Head and Neck Society.

### Delphi exercise

Following the Consensus Day, ratification by the steering committee led to clarifications of the wording for 12 statements, the addition of a single statement (to accommodate HPV-positive as well as HPV-negative disease) and the removal of six statements due to duplicated content.

#### Responses

**Fig. 1:**
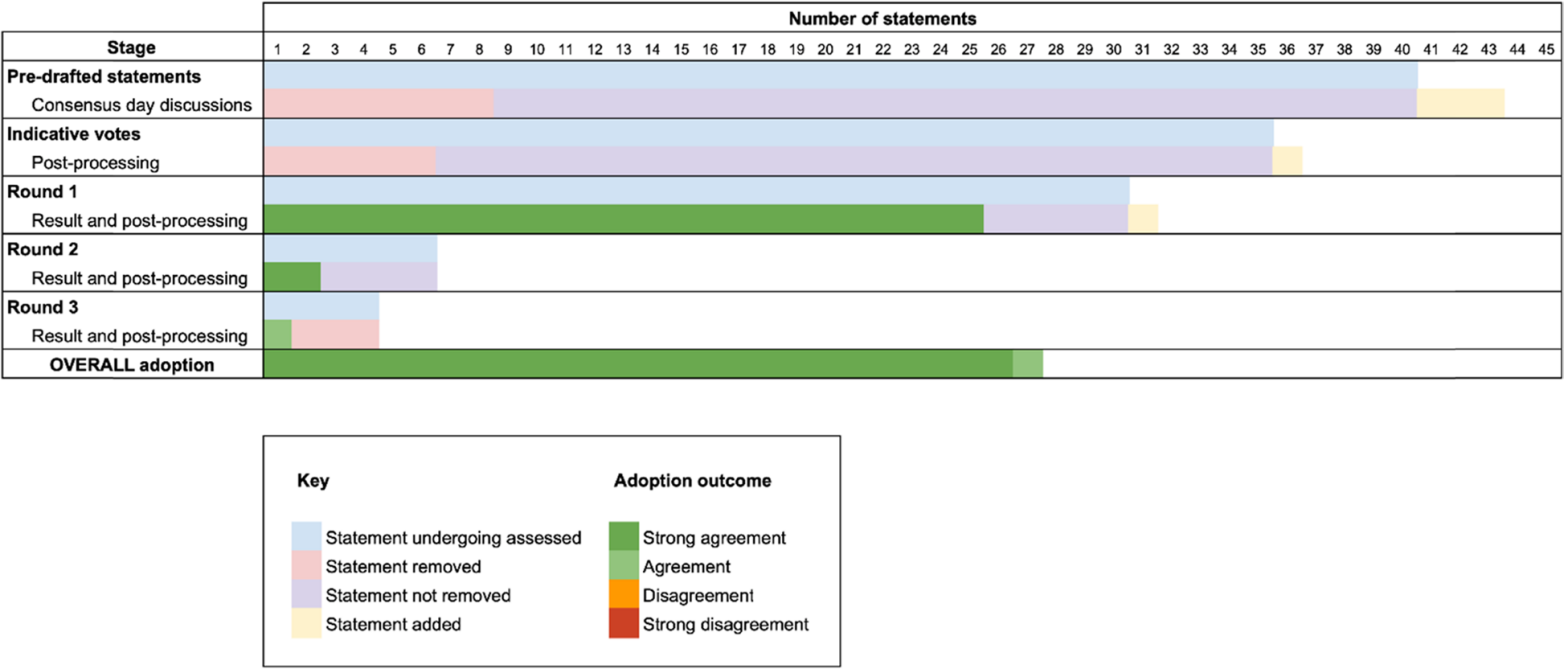
Graphical summary of outcomes from the multi-stage meta-consensus exercise

Results from the 3 rounds of Delphi are presented in Fig. 1. The overall response rate was 98% (*n* = 57/58) for each of the three rounds. A response to every statement was required in order to submit the Delphi form. However, for 9 instances (0.4% of 2280 total responses), requests were made to abstain from the vote for that statement as a consensus from within that MDT could not be reached.

#### Changes between rounds and adoption

A single statement was added after round 1 for round 2, incorporating ‘consider’ phrasing as per the a priori methodology. No further statements were added for round 3. Strong agreement was reached for 27 statements (*n* = 25 in round 1, 2 in round 2 and none in round 3 and a single statement only reached agreement at the end of round 3. No agreement could be reached for 3 statements and none reached thresholds for any level of disagreement. Consequently, following the 3-round Delphi process, 28 statements were re-distributed to the representatives of UK HN MDTs for endorsement and were subsequently incorporated into the 6th edition of the ‘United Kingdom National Multidisciplinary Guidelines for Head and Neck Cancer’.

## Discussion

### Summary of findings

This was a novel initiative pioneering a multi-stage meta-consensus methodology, building on presentations of the best available evidence, to generate multidisciplinary recommendations for the management of a controversial disease. The Consensus Day involved 61 health professionals who facilitated the delivery of the evidence-based presentations and breakout discussions, with 227 attendees helping to generate 35 draft statements, prior to a Delphi exercise directly involving 58 MDT contacts, with many other members of head and neck MDTs also consulted. The entire exercise was delivered by a voluntary steering committee and, though not intended, generated a net profit for the parent organisations.

Following the Consensus Day, 83% of statements achieved ‘strong agreement’ after the first round of the modified Delphi process. Ultimately, only 3 out of the 31 statements considered did not reach consensus according to our prespecified thresholds. It is likely the inclusive methodology employed by the Consensus Day, encouraging input from myriad UK health professionals, ensured that, by the time of the subsequent Delphi consultation exercise, there was already widespread support for the statements generated during the opening round. Support may have been further garnered by the widespread participation in the National Audit, which saw data submitted from 56 centres representing 38 of 58 UK HN MDTs [18].

### Research in context

There are a number of alternative guidelines available for management of HNSCCUP which are summarised in table 1 [4–9]. However, the authors chose to develop this bespoke methodology for the production of these latest guidelines due to a relative paucity of available evidence and to maximise the potential for adherence to the resultant output. We accept that adherence will be hard to measure and is beyond the scope of this project.

**Table 1:** Summary of commonly used guidelines in the management of head and neck squamous carcinoma cancer of unknown primary and their methodology

Publishing/ endorsing organisation(s)	Organisation(s) abbreviation/ acronym	Year published/ updated	Publishing country	Journal	Title	Publication dedicated solely to HNSCCUP	Number of recommendations/ statements dedicated to HNSCCUP	Methodology	Patient/ public inclusion
*United Kingdom National Multidisciplinary Guidelines for Head and Neck Cancer*^b^	*UK MDT*	*Anticipated 2022*	*UK*	*Journal of Laryngology and Otology*	*Management of head and neck squamous cell carcinoma of unknown primary (HNSCCUP): United Kingdom National Multidisciplinary Guidelines*	*Yes*	*28*	*A National Consensus Day including 227 multidisciplinary attendees generated draft statements based on systematic reviews and expert presentations.* A priori *methodology published.* *Consultation included a National Delphi Exercise to gauge consensus for inclusion of recommendation.* *Peer reviewed.*	*Patient experience presentation delivered at National Consensus Day prior to generation of draft consensus statements.*
British Association of Head and Neck Oncologists standards	BAHNO	2021	UK	Journal of Oral Pathology and Medicine	British Association of Head and Neck Oncologists (BAHNO) standards 2020	No	5	20 multidisciplinary authors took reference from national published guidance to inform the recommendations. No a priori methodology published. No wider consultation following development was declared. Peer reviewed.	Nil
National Comprehensive Cancer Network	NCCN	2021	USA	–	Head and Neck Cancer Guidelines (Version 1.2022)	No	4 flowchart and roughly 9 statements	Developed by a multidisciplinary panel of 36 experts and 2 support staff. A priori methodology published. No wider consultation following development was declared. No Peer review prior to publication.	Inclusion of a patient advocate on the panel is encouraged but their involvement is not explicitly declared.
American Society of Clinical Oncology	ASCO	2020	USA	Journal of Clinical Oncology	Diagnosis and Management of Squamous Cell Carcinoma of Unknown Primary in the Head and Neck: ASCO Guideline	Yes	33	15 multidisciplinary authors formed an Expert Panel who assessed 100 relevant articles from systematic reviews. A priori methodology published. Consultation included an open public comment period of two weeks. Peer reviewed.	Draft recommendations were open to public comment on signing a confidentiality agreement.
European Head and Neck Society, European Society for Medical Oncology, and European SocieTy for Radiotherapy and Oncology	EHNS/ESMO/ESTRO	2020	Europe	Annals of Oncology	Squamous cell carcinoma of the oral cavity, larynx, oropharynx and hypopharynx: EHNS-ESMO-ESTRO Clinical Practice Guidelines for diagnosis, treatment and follow-up	No	Roughly 250 words of prose	20 multidisciplinary authors assessed evidence and wrote guidelines. A priori methodology published. No wider consultation following development declared. Peer reviewed.	Nil
National Institute for Health and Care Excellence	NICE	2018	UK	–	Cancer of the upper aerodigestive tract: assessment and management in people aged 16 and over	No	8	A multidisciplinary advisory group forms a writing committee to generate draft statements based on ‘evidence reviews’. A priori methodology published. Consultation included release of draft guidelines for input from registered stakeholders. Peer reviewed ‘may occasionally be considered’ but not explicitly declared.	Included in the guideline generating committee.
United Kingdom National Multidisciplinary Guidelines for Head and Neck Cancer^a^	UK MDT	2016	UK	Journal of Laryngology and Otology	Investigation and management of the unknown primary with metastatic neck disease: United Kingdom National Multidisciplinary Guidelines	Yes	10	Five multidisciplinary experts HNC. No a priori methodology published. No wider consultation following development was declared. Peer reviewed.	Nil

^a^guidelines endorsed: by the British Association of Otorhinolaryngology-Head & Neck Surgery (ENT UK); the British Association of Oral and Maxillofacial Surgeons (BAOMS); the British Association of Plastic, Reconstructive and Aesthetic Surgeons (BAPRAS); British Association of Head and Neck Oncologists (BAHNO); The Royal College of Pathologists (RCPath); The Royal College of Radiologists (RCR); and the British Association of Endocrine and Thyroid Surgeons (BAETS)

^b^endorsing organisations to be confirmed prior to publication

#### Alternative guidelines of management of head and neck Cancer of unknown primary

The previous iteration of the United Kingdom National Multidisciplinary Guidelines for Head and Neck Cancer (that this project replaced) were drafted by five multidisciplinary experts HNC, with backgrounds in speech and language therapy, oncology and ENT surgery. They were then reviewed and adopted by representatives from a variety of multidisciplinary stakeholder organisations [4, 19]. These guidelines considered the contemporary evidence but did not declare any systematic methodology for identification or appraisal. Further, with a limited number of professionals involved in their generation, without further consultation, the guidelines risk being unacceptable to many centres across the UK where practice may be constrained by available resources and services.

National Institute for Health and Care Excellence (NICE) Guidance 36, for Cancer of the upper aerodigestive tract (2018), underwent NICE’s standard rigorous process for guidance development [2, 5]. It goes further to specify how the evidence was appraised: identifying a topic, agreeing its scope amongst stakeholder organisation and then agreeing review questions. The literature was then searched to produce ‘evidence reviews’ which were then ‘considered by a committee made-up of practitioners, professionals, care providers, commissioners, those who use services and family members or carers’. Draft guidelines were produced by the committee and sent to stakeholders for comment before being revised and sent to the senior ‘Guidance Executive’ before publication. Whilst this also goes further in attempting to engage more widespread opinions, response rates from the consultation process tend to be low. Additionally, the ‘consider’ phraseology adopted by NICE (and replicated here) has drawn criticism for being too broad in scope, covering recommendations that may lack sufficient evidence to reach an ‘offer’ threshold, but also those where the intervention may be thought of as optional or as only occasionally appropriate.

Guidelines from the European Head and Neck Society (EHNS), European Society for Medical Oncology (ESMO), and European SocieTy for Radiotherapy and Oncology (ESTRO)8 and from the National Comprehensive Cancer Network (NCCN) have similar development methodology development to the NICE recommendations in that they cover all of head and neck cancer (not just HNSCCUP specifically) and rely on a limited multidisciplinary panel of experts in their initial development [9, 20].

The American Society of Clinical Oncology (ASCO) published 33 recommendations in 2020.6 Guidelines were generated by an expert multidisciplinary panel who had reviewed systematic reviews, including 100 articles, and then rated the certainty of the evidence and the strength of the recommendation using GRADE (Grading of Recommendations Assessment Development and Evaluation) methodology.1 Importantly, they go further in seeking widespread consensus by releasing the draft recommendations for open comment from the public. This process allows any individual to give input, but responses are not required or expected and any feedback must be approved by a Clinical Practice Guideline Committee before adoption. Their effort to be inclusive of lay members, including patients, are to be commended, though it is acknowledged that vocal minorities may be overrepresented without a comprehensive countrywide framework for seeking and processing feedback in place.

An area for potential improvement in all the methodologies explored here, is the engagement of a greater number of stakeholders giving more representation. Particularly, seeking more input from the multidisciplinary team (MDT) members who are actually delivering the care day-to-day in the majority of UK centres, not just a selection of ‘experts’ who may not have an informed picture of the limitations of delivering care outside of tertiary referral centres. The methodology described herein aimed to address these potential deficiencies through the Consensus Day and Delphi Process. Without this comprehensive engagement, recommendations for this challenging and controversial condition risk being admirably aspirational, but adoption may be limited if they do not garner sufficient buy-in from individual units, and they may be unachievable, depending on local service arrangements. Buy-in is essential if guidelines are to be adopted and, ultimately, to achieve their aim of influencing clinical practice. Perhaps the first key step of any new guideline is to homogenise standard care to reduce the potential for health inequalities across a healthcare system. Through engagement with multiple stakeholders at the draft statement stage, and then by adapting the threshold for consensus agreement in the Delphi stage, the final guidelines may be titrated to the resources and aspirations of the individual healthcare system, without unnecessarily entrenching suboptimal practice from being too conservative.

### Limitations

Limitations to this initiative are acknowledged. Firstly, attendance at the Consensus Day was self-selected, giving the potential for disproportionate representation from one or more stakeholder groups. Secondly, during the Delphi exercise, the contact was asked to record the consensus view of their MDT. However, the level of true consultation cannot be gauged or recorded using this methodology, and so responses may be biased towards the specialty viewpoint on the contact (ENT in this instance). Thirdly, organisation of the hybrid consensus event was relatively labour intensive, particularly corralling the contributors to deliver their contributions on time. This work fell largely to the clinical research fellow and their supervisor and was essential to ensure successful delivery. Fourthly, the steering committee did not ask about conflicts of interests, and so the process may have been susceptible to individual biases. Finally, the degree of patient consultation was limited. However, patient views were considered at multiple points: a ‘patient experience’ interview was delivered as the opening presentation at the Consensus Day to provide qualitative data; and the National Audit was designed to collect quantitative data to better consider the timeline for interventions in the patients’ diagnostic pathway from all across the UK.

### Implications for future work

A key aim of this methodology was to generate guidelines that would see meaningful adoption and improvements in patient care. As such, an implementation exercise is planned to use Evidence-Based Co-Design (EBCD) methodology to optimise the patient pathway in a regional cancer network, as a template for national adoption [21].

## Conclusions

The described methodology demonstrated an effective and inclusive multi-phase strategy for the development of national practice recommendations for the management of HNSCCUP, a relatively uncommon and controversial disease. The initiative achieved widespread engagement, including a well-attended Multidisciplinary National Consensus Day, comprehensive National Audit, and a Delphi process including representation from all 58 UK Head and Neck MDTs. This may serve as a model for future guideline development for controversial or rare conditions where there is a paucity of available evidence or where there is significant variability in management practices across a health service, and where widespread buy-in for the resultant output is desirable.

## Supplementary Information

**Additional file 1.**

## Data Availability

The datasets used and/or analysed during the current study are available from the corresponding author on reasonable request.

## Abbreviations

AOT: Association of Otolaryngologists in Training
ASCO: American Society of Clinical Oncology
EBCD: Experience-Based Co-Design
EHNS: European Head and Neck Society
ESMO: European Society for Medical Oncology
ESTRO: European SocieTy for Radiotherapy and Oncology
HNC: Head and Neck Cancer
HNSCCUP: Head and Neck Squamous Cell Carcinoma of Unknown Primary
MDT: Multidisciplinary Team
NICE: National Institute for Health and Care Excellence
PET-CT: Positron emission tomography/Computed tomography
UK: United Kingdom
