# Supplementary material for: Methodology for the development of National Multidisciplinary Management Recommendations using a multi-stage meta-consensus initiative
Source: BMC Med Res Methodol. 2022 Jul 11;22:189. doi: 10.1186/s12874-022-01667-w (PMC9275134; doi:10.1186/s12874-022-01667-w)
Supplement: Supplementary file 1 — Additional file 1. [file 12874_2022_1667_MOESM1_ESM.pdf]

An ENT UK H&N Society Initiative

HNSCCUP

Multidisciplinary Consensus Process

Results from indicative vote to final Delphi

1: Investigations before diagnostic surgery for clinically suspected HNSCCUP

No.	Statement	Indicative vote	Round 1	Round 2	Round 3	Outcome
1a	Offer all patients with clinically suspected HNSCCUP ultrasound guided sampling as a first-line investigation to diagnose cervical metastasis of SCC, which must include p16 and/or HPV subtyping and ancillary tests	[98.1%][a]	[96.5%]	-	-	Strong agreement
1b	Do not offer open biopsy to patients with a neck lump as a first line investigation to diagnose cervical metastasis	[98.1%][b]	[98.2%]	-	-	Strong agreement
1c	Offer all patients with clinical suspicion of HNSCCUP a concurrent MRI and PET-CT as first-line cross sectional imaging investigations	[77.1%][b]	[82.5%]	-	-	Strong agreement
1d	Consider image enhancement technology (including narrow band imaging) as an adjunct to white light endoscopy in the examination of all patients with clinically suspected HNSCCUP	[96.2%][a]	[89.5%]	-	-	Strong agreement
1e	Refer all patients with clinically suspected pathologically confirmed HNSCCUP to a core member of head and neck MDT for further investigations	[93.4%][a]	[98.2%]	-	-	Strong agreement

[a] n=106, [b] n=105

Comments on Section 1

- 1c is a game changer for many many units and would certainly improve the pathway - but it lacks evidence really to support the costs of instituting this. Ask our US colleagues. Even Chris Holsinger says on the recent board meetings on line that most US surgeons use CT, based on cost, and struggle with MR and PET CT unless absolutely indicated.
  To me - this is the single biggest gain from the consensus day and I would suggest we look at a prospective cost analysis trial for this question.
- 1c - depends on level of clinical suspicioun. Pure cystic masses this may be overkill
- All patients with a neck mass should be seen in a one stop neck lump clinic with access to same day core biopsy - results should be available within 3 working days. PET-CT should be done as a second line investigation with confirmed pathology. First line PET-CT on the basis of clinical history and examination alone will result in excess radiation, unnecessary investigation of incidental findings, and is a waste of precious resource.
- Question 1a - do ancillary tests cover EBV in HPV/p16-ve samples?
- I would suggest 1d is 'offer' rather than 'consider'?
- Issue of staging chest not explicit.
- Contrast CT comparible to MRI as initial cross sectional imaging modality, is more quickly accessible, and suffers from less movement artefact. NBI is a useful diagnostic tool in dysplastic/ early invasive lesions but I have not seen data validating it's use in this population of patients the majority of whom have a tongue base or tonsil occult primary
- We CT and don't have MRI in our cup pathway. Given CT has pick up rate for primary and avoids pet which is strained service then not sure justify doing 2 together and not sure need for mri above ct and pet
- Should Initial biopsy not be stipulated to be a core biopsy?
- CT neck thorax is adequate rather than MRI I think offer CT neck thorax or MRI neck CT thorax and consider concurrent PET CT
- I would do a CT rather than an MRI as we do not have easy access to timely MRI. Also the patient is this lets us image the thorax for staging. In our area we do not have the resource (radiologist or scannners) to do both on every patient.
- Avoid the Use of NBI as this is only applicable to Olympus (should not favour one company over another). The generic term should be Virtual ChromoEndoscopy (VCE) that also takes into account SPECTRA/SPIES, PIET and NBI
- 1c- concerns about ARSAC permissions in a non confirmed cancer
- PET scans are not available within a few days in our unit (district general hospital) as it is carried out in another larger unit in the region. We usually find a delay of 2 to 3 weeks for our patients to get a PET scan from request. Therefore an MRI scan of the oropharynx and a staging CT neck and chest are obtained as first line by our MDT (both requested as soon as the Ultrasound scan and/ or clinical examination suggests a pathological node without an obvious primary). The CT and MRI scans would usually identify the vast majority of primaries. If no primary is identified on CT and MRI scans, then we would request a PET scan.
- 1c in our opinion should be consider not offer
- Ic consider local radiologists views as has an impact potentially on service
- All members of our MDT advocate upfront core not FNA given requirement/accuracy of P16 analysis. We feel strongly this should be advocated in the consensus. This is clinically the correct thing to do and consensus statement should drive service development if not universally available.
  New national standards advocate same day MRI in a one-stop clinic. We already have this available and this enables us to refer for PET OR CT the day after clinic depending on if a primary is identified on MRI.
- 1c This would depend on the definition of clinical suspicion and the experience of the clinician ordering the investigations
- I think 1e needs to be changed, because it doesn't make much sense to have a suspected cancer if it is pathologically confirmed. It should probably read "pathologically confirmed mets with clinically unknown primary" or simply "pathologically confirmed HNSCCUP"

2: Diagnostic surgery for clinically suspected pathologically confirmed HNSCCUP

No.	Statement	Indicative vote	Round 1	Round 2	Round 3	Outcome
2a	Perform all radiological investigations aiming to identify the primary site prior to discussion at head and neck MDT and diagnostic surgery	[93.0%][a]	[94.7%]	-	-	Strong agreement
2b	Offer nasopharyngeal biopsies when the cervical node sampling reveals Epstein-Barr virus positive metastasis	[93.0%][b]	[98.2%]	-	-	Strong agreement
2c	Do not offer biopsies of clinically and radiologically normal upper aerodigestive tract mucosa. This excludes tonsillectomy or tongue base mucosectomy	[93.0%][b]	[91.2%]	-	-	Strong agreement
2d	Offer ipsilateral tonsillectomy (rather than incisional biopsy) to all patients	[80.0%][a]	[89.5%]	-	-	Strong agreement
2e	Consider contralateral tonsillectomy (rather than incisional biopsy) in all patients	[94.7%][b]	[100%]	-	-	Strong agreement
2fi	Offer ipsilateral tongue base mucosectomy to all patients	[73.5%][c]	[71.9%]	[59.6%]	[61.4%]	No agreement
2fii	Consider ipsilateral tongue base mucosectomy in all patients	-	-	[89.5%]	-	Strong agreement
2g	Consider contralateral tongue base mucosectomy for all patients	[87.6%][c]	[78.9%]	[80.7%]	-	Strong agreement
2h	The term 'Ipsilateral oropharyngeal MALTectomy' is appropriate to represent the removal of the palatine tonsil and the lingual tonsil on the affected side	[68.8%][d]	[59.6%]	[47.4%]	[49.1%]	No agreement
2i	Perform tongue base mucosectomy using one of the following transoral techniques, when indicated: endoscopic, microscopic or robot-assisted	[97.4%][b]	[96.5%]	-	-	Strong agreement

[a] n=115, [b] n=114, [c] n=113, [d] n=109

Comments on Section 2

Round 1

- I think you need a set of statements that will have an effect. 2c is fairly meaningless. We know that random biopsies yield little - but they don't cause harm. The statement should read to recommend tonsillectomy and base of tongue mucosectomy over random biopsies of these sites. Taking random biopsy of the PNS or PF doesn't cause any harm.
- 2d needs some thought. In experienced hands - most tonsil tumours are visible under GA and should have an incisional biopsy not tonsillectomy especially if transoral surgery is to be considered. We don't want a statement encouraging tonsillectomy over a really considered EUA and biopsy of abnormal looking tissue. These occur a lot and may be MRI / PET CT negative.
- Tongue base mucosectomy can be done without endoscope,microscope or ROBOT
- Mucosectomy is clearly going to be cotroversial here and approach will depend on MDT feelings about accepting P16/HPV as being from oropharynx. I suspect there will be divergent opinions here
- The evidence based that tongue base mucosectomy in p16+ve patients changes treatment or outcomes for the better i.e. safe de escalation, needs to be clearer - I would suggest that it should only be carried out in the contest of a trial.
- 'Oropharyngeal MALTectomy' is unnecessary - it's confusing for patients and clinicians
- 2d: I think bilateral tonsillectomy (if you cannot palpate a lesion to biopsy) is appropriate initially, rather than ipsilateral. 2f: if it not clear if there is an option for palatine tonsils first, then BOT? 2g; is there an option for 2/3 BOT mucosectomy.
- MALTectomy not very popular. New descriptions can be confusing
- Mucosectomy and bilateral tonsillectomy represents a significant oropharynx intervention with associated cicatrisation and will affect function in this swallowing critical zone and may contribute to late radiation associated dysphasia. It has not been demonstrated to improve survival outcomes. The tongue base is a midline structure with bilateral lymphatic drainage and therefore ipsilateral mucosectomy/ tonsillectomy to mitigate has a doubtful rationale.
- Think should have add on about straight forward cases or in cases with no uncertainty. This statement just puts a lot of pressure on referring surgeons not to bring cases to mdt and although that’s ok for most there is some cases where earlier discussion might be helpful and statement needs to reflect that and allow some wiggle room
- Malt just reminds me of malt lymphoma
- I'd like clear evidence before recommending increasingly morbid hunts for the primary ahead of radiotherapy, if excellent imaging is negative. The relatively low rates of relapse at primary site implies this is unnecessary.
- It should be more explicit regarding sequencing of removal of tonsils and then TBM if negative?
- Our OMFS colleagues commented on the term 'Lingual Tonsil' . This is indeed MALT tissue in the tongue base but apparently OMFS think this is the tissue laterally on the tongue what is better known as the foliate papillae. Should Lingual Tonsil be define clearly for OMFS?
- 2h) A better term might be 'ipsilateral palatine and lingual tonsillectomy'
- We do bilateral tonsillectomy up front
- I agree that tongue base mucusectomy is often indicated, but I answered 'no' to 2f as not indicated for all patients. Same can apply to tonsils in certain patients (eg surgical v high risk, already have SCC and P16 status on a node core biopsy and oncologists happy to give RT without a definite primary). As bilateral tonsillectomy with tongue base mucusectomy v painful, we will often do bilateral Ts with BOT biopsies and consider mucusectomy if path negative on Ts and BOT Bxs. Often primary is identified without the need for full mucusectomy. we do discuss all options with patients though.
- Everyone in the H&N community is used to the terms 'Bilateral diagnostic tonsillectomy and tongue base mucosectomy' so our MDT sees no advantage in introducing a new term in the H&N vocabulary 'oropharyngeal MALTectomy'
- MALT term is confusing and not clear, better to stick with anatomical description as risk of it otherwise meaning different things to different people. In elderly/ frail patients mucosectomy is a significant undertaking for them so need to consider this and not be too didactic
- Comment was made on 'referral to unpublished MOSES trial'

- It seems bizarre that 73% advocated ipsilateral but 87% advocated contralateral. I'm guessing this is due to offer v consider
- I find it unclear what is advocated if a primary is identified clinically at panendoscopy prior to 'MALTectomy'. I would favour biopsy (+/- frozen section to confirm) as these patients are likely highly suited to PATHOS and extensive oropharyngeal surgery would deny them this opportunity.
- 2d and 2e Offer bilateral tonsillectomy except when an obvious tonsil primary tumour is identified where tonsil biopsy may be appropriate
- 2d- unless there is an obvious tonsil cancer found during EUA
- 2h- MALTectomy likely to cause confusion
- 2a should probably define what is considered 'all radiological investigations', or mention there should be a local agreement as to what that means
- 2c: do not offer us too strong, depends on availability and timeliness of mucosectomy.
- We would have agreed 2f if it were Consider
- 2f should be consider. In the real world a mucosectomy is not readily available and will add to workload to subspecialist surgeons and delay patients unnecessarily

Round 2

- We should at least offer ipsilateral tongue base mucosectomy for unknown primary cancers but not all surgeons perform this or have the service. The vast majority of unknown primary will be identified with TBM and bilateral tonsillectomy. Contra lateral TBM though has a lower yield with more morbidity / pain. MALTectomy is a new term and unsure it fits the current description for TBM.
- no need to add a new terminology in my view
- 2fi & 2fii: I would suggest that this can be discussed with the patient, although not necessarily recommended.
- 2g: I think bilateral tonsillectomy and bilateral mucusectomy at one sitting to much. Possible exception would be a patient who has previously had a tonsillectomy.
- 2h: would add confusion rather than clarification in practice (although may be good in theory!)
- If question 2h remains un-agreed, why not discard it, as it represents a change in terminology rather than a change in clinical practice.
- 2fi may read better if it includes "HPV related SCC confirmed on neck node needle sampling".
- The evidence presented was very strongly in favour of this diagnostic intervention - for HPV related disease, less so for HPV negative disease.
- If we adopted this statement as "offer" it would certainly improve the access to this intervention for patients and would be supported by the presented evidence.
- There was some confusion in the meeting - suggestions that offer ipsilat and consider contralat BoT mucosectomy would result in 2 procedures, or even 4 if we had offer ipsilat tonsillectomy and consider contralat tonsillectomy as well. It would be ideal to somehow get the notion across that the ipsilateral tonsillectomy and BoT mucosectomy have the highest level of pick up - "considering" the contralateral diagnostic interventions (at the same time as the ipsilat) then allows an individual patient decision to be made - eg for an elderly patient, ipsilateral intervention alone may be appropriate.
- I'm not sure what we are hoping to gain from the term MALTectomy - unless we are suggesting that all patients undergo the tonsillectomy with the BoT mucosectomy at the same initial sitting.
- 'Ipsilateral oropharyngeal MALTectomy' is an unnecessarily complex term - will complication communication
- Mucosa associated lymphoid tissue' is used to describe small aggregates of submucosal lymphoid tissue that are not otherwise named, not lymphoid structures like the lingual and pharyngeal tonsil.
- Agree with comments that this extra nomenclature has potential to confuse, and is not necessary
- I Think bilateral tonsillectomy that accompanies tongue base mucosectomy is likely to have significant swallowing implications for some without data demonstrating that it influences prognosis. It's use should therefore be restricted to trials designed to demonstrate an oncological advantage. I am in two minds about an ipsilateral mucosectomy as I regard the posterior third of the tongue (oropharynx tongue) as a midline structure from the standpoint of lymphatic drainage. However, there may be specific circumstances where a lateralised lingual tonsillar primary/ lower pole tonsil seems a distinct likelihood e.g equivocal FDG PET-CT findings or cross sectional imaging asymmetric change.
- MALTectomy is introducing more terminology that is confusing. I think the evidence does not go beyond consider at the moment
- I'd agree, isolateral MALTectomy is likely to be confusing
- Need to carefully consider evidence before suggesting mucosectomy for all unknown primaries, esp HPV negative. For example, recent papers like Kubik etal from Pittsburg and Copenhagen do not show any advantage for mucosectomy in HPV negative patients.

- 2fi and 2fii - our MDT is divided, but the majority consensus is to 'offer ipsilateral' BOT mucosectomy (irrespective of p16 status of neck core biopsy) and 'consider contralateral' BOT mucosectomy as most of the primary has been demonstrated to be identified in the ipsilateral/midline BOT mucosectomy specimen.
- We would do Pan-endoscopy (including nose), EUA+ Biopsy, bilateral tonsillectomy and if all negative bilateral tongue base mucosectomy. I am not sure which option covers this
- TBM has significant morbidity so consider in frail patients otherwise generally if patient is fit then reasonable to offer. We do not feel the term MALTectomy is useful or clear and risks introducing confusion so would be reluctant to use it, better to stick to accurate anatomical descriptions of sites.
- Comment on 2fi - with caveat that would assume should offer total TBM in all patients if no primary in tonsils found/ and oncologists would still wish to irradiate tongue base as only ipsilateral tongue base addressed

Round 3

- 2fi needs to be consider
- Offer ipsilateral tongue base mucosectomy to all patients' is too strong and too vague. Suggest adopt the language of NICE for this recommendation i.e. 'Consider' ipsilateral TBM in all patients.... (NICE also use 'must' and 'should' which equate to 'beyond reasonable doubt' and 'on the balance of probabilities' respectively)
- ‘Ipsilateral oropharyngeal MALTectomy’: don't see the need for new terminology plus from a purists perspective this would require removal of the the entire oropharyngeal mucosa including sections of the ipsiltateral palate and posterior pharyngeal wall
- We think 2fi should be 'consider' and our oncologist felt that 2h should be kept as simple as possible, and not adding additional terms.
- 2fi I think this statement should be removed as there has already been agreement on "Consider mucosectomy..."
- 2h I don't see where the confusion would come from as it is like any other term we use and it would help not having really long operation titles.
- For 2Fi :we suggest 'consider'. We have seen small contralateral BOT lesions. Knowing all the BOT tissue has been removed offers reassurance to the MDT that this site has been excluded for an unknown primary.
- For 2H : agree on the basis that A MALTECTOMY is different to doing a separate Tonsillectomy and BOT mucosectomy as the MALTECTOMY takes the Glosso Tonsil Sulcus Tissue and this band of tissue can be missed in above procedures.
- The 'offer ipsilateral tongue base mucosectomy' option now becomes slightly out of context without being followed by the 'offer contralateral tongue base mucosectomy option'. We only do bilateral tongue base mucosectomies.
- 2fi: The problem is "offer to all". I would say "consider" because there may be a reason not to offer.
- 2h: I put this to our MDT and they felt it confusing (because of MALToma)
- MALTectomy - There is no need to introduce a new terminology which will not change practice but more likely to confuse others (GPs for eg)
- The term is overly complicated and the feeling is it is not needed. As stated previously we would consider mucosectomy in all patients so that term probably fits better than offer.
- Offering ipsilateral TBM and bilateral tonsillectomy is satisfactory for HPV Unknown primary but TBM may not yield much in HPV negative disease and only adds morbidity.
- Maltectomy is not a universally agreed terminology.
- I have consistently ticked agree for 2f as we believe the evidence is strong enough in favour of BOT mucosectomy - but the statement could be clarified further by specifying for proven HPV related neck disease. The evidence for HPV negative disease was not definitive on the day, in keeping with many surgeons' anecdotal experiences. For HPV neg "consider" may be a more appropriate statement. "In patients with appropriate transoral access" could be a useful addition too, as for some patients with poor access, the procedure is difficult to perform and remove all lymphoid tissue - in these situations a poorly performed mucosectomy may lead to more harm (both pain and lack of definitively negative specimen) than good.
- I have considered MALTectomy and refer to my previous comment - I am not sure what we are trying to achieve with it. The greatest gain in the UK would be to promote appropriate BOT mucosectomy use. Whether to perform this with a tonsillectomy can be debated. I don't see anything wrong with a unit performing pharyngoscopy, tonsillectomy, access for BoT mucosectomy, and if negative then sending to another unit to perform the BOT mucosectomy if they do not do so. This would be a step forward than not offering the HPV related patients a mucosectomy.

- I think my issue with MALT ectomy is that is avoids the notion of BOT mucosectomy being a specialist diagnostic intervention (by linking it with tonsillectomy in one word) - which it is given the necessary MDT discussions that go with it and overall management plans.
- I'm not sure why anyone would feel strongly enough to object to the term "MALTectomy" - it is logical!
- 2fi - No - We would suggest bilateral TBM

3: Surgical management of patients diagnosed as HNSCCUP

Unless otherwise specified, the HNSCCUP patients referred to in this session are assumed to have undergone an adequate diagnostic work-up, as per their MDT, and are due to commence treatment as a true HNSCCUP.

No.	Statement	Indicative vote	Round 1	Round 2	Round 3	Outcome
3ai	Consider ipsilateral tonsillectomy and tongue base mucosectomy (ipsilateral oropharyngeal MALTectomy) and ipsilateral neck dissection in HPV positive HNSCCUP with a single node less than 3cm and with no radiological evidence of extranodal extension	[84.1%][d]	[87.7%]	-	-	Strong agreement
3aii	Consider ipsilateral tonsillectomy and tongue base mucosectomy (ipsilateral oropharyngeal MALTectomy) and ipsilateral neck dissection in HPV negative HNSCCUP with a single node less than 3cm and with no radiological evidence of extranodal extension	-	[68.4%]	[75.4%]	[77.2%]	Agreement
3b	Consider adding regular cross sectional imaging to regular clinical examination for post-treatment surveillance of patients treated with surgery as a single modality, following bilateral oropharyngeal MALTectomy and pN1 disease with no extranodal extension	[84.8%][a]	[84.2%]	-	-	Strong agreement
3c	Consider neck dissection prior to treatment in HPV negative HNSCCUP undergoing radical radiotherapy with advanced disease unsuitable for concomitant chemotherapy	[84.3%][c]	[93.0%]	-	-	Strong agreement
3d	Consider neck dissection prior to radiotherapy +/- chemotherapy in HPV -ve HNSCCUP patients with N3 neck disease	[87.9%][b]	[89.5%]	-	-	Strong agreement
3e	Consider contralateral staging neck dissection where there is no clinical or radiological evidence of disease to allow omission of contralateral neck radiotherapy	[62.3%][e]	[38.6%]	[35.1%]	[38.6%]	No agreement

[a] n=92, [b] n=91, [c] n=89, [d] n=88, [e] n=61

Comments on Section 3

Round 1

- These are all consider statements and represent the nuances of MDT discussions. They don't have the weight to produce practice change that the offer statements do. None of these deserve Offer status - agree with that.
- I don't see why 3b shold be different from other disease stages
- 3e - difficult one. I think there is a balance to be struck. Is the morebidity from your neck dissection worse than that from the additional RT field?? Controversial
- How about '3b consider US surveillance' - CT/ MRI would be a waste of resource
- 3e surgical contralateral neck staging - too morbid
- Pittsburgh study showed a pick up rate of only 13% with tongue base mucosectomy for HPV negative CUP . There is no good evidence to justify contralateral neck dissection to allow omission of contralateral neck RT
- Late radiation dysphagia and other late toxicities have a strong association with surgery. Therefore the use of surgery should be reserved for those patients with a poor prognosis with non- surgical management. This includes those with HPV and EBV negative disease and recent SEER data suggests those with locally advanced (T3/T4) HPV +ve disease
- Strongly disagree with therapeutic neck dissection at same time as a staging diagnostic procedure. If positive, which is about 1/3, then subjects patient to multimodality treatment with surgery then radiotherapy which for a excellent prognostic cancer comes with morbid toxicity effects and is unnecessary treatment as seen in PET NECK
- 3b - not sure evidence on effect on outcomes to suuport this? 3e. We would believe modern ct and pet and us neck as enough staging to exclude contralateral disease and allow ipsilateral treatment
- Omission of contralateral neck RT is a valid option for patients without a staging neck dissection
- for statement 22 - in HPV-ve disease the oropharynx is not necessarily the most likely primary site, so the added morbidity may be difficult to justify. for statement 20 - would follow PET neck protocol for statement 22 - concerned about the significant increase in morbidity associated with bilaterla neck dissection followed by bilater neck radiotherapy, if disease is discovered in contralateral neck. FO T0 tumours, our practice is to irradiate unilateral neck only, with salvage treatment at relapse if needed.
- 3aii some confusion in- HPV negative HNSCCUP-role of TBM and Tonsillectomy+/- ND
- 3a1 we would do bilateral Ts
- 3b we would do single post treatment PET, further imaging only if indicated
- We would consider a neck dissection for 3ai, but would usually try to find the primary first as otherwise neck dissection unnecessary as RT needed anyway.
- 3b We do 3-4 month PET, same as the RT and CRT patients
- 3ai- this is suggesting that a neck dissection should be performed with the MALTectomy where this is still part of the diagnostic process. Surely this is one option. Treatment with neck dissection may not be required if the patient were to choose RT. Also - this complicates things of a primary is found and further resection with a messed ligation is needed.
- For investigation of an unknown primary, as a bare minimum we would perform a bilateral and not just an ipsilateral tonsillectomy. I have had a couple of patients presenting with bilateral tonsil SCC. Traditionally, when our oncologists say the radiotherapy given to the neck will cover the base of tongue, a tongue base mucosectomy has not been performed in order not to delay treatment. In addition, limited access to the robot means that we cannot do a tongue base mucosectomy at the same sitting as an EUA with tonsillectomy.
- Do not agree with MALTectomy term, 3c/d a bit controversial ? conflicts with PET neck trial data
- 3b - whilst regular imaging, may have a place here I feel this statement is too vague to be useful and without evidence base to support the implications on service delivery. What imaging? What does regular mean? What evidence do we have to support this over close clinical surveillance?
- 3e - whilst this seems a potentially attractive proposition we do not believe there is sufficient evidence to support this in terms of improved patient morbidity/experience. This surely needs to be subject to RCT before adopting into national guidance

- 3ai and 3aii Whilst the neck disease would be treated with neck dissection we would recommend bilateral tonsillectomy prior to this. These statements would stand if "ipsilateral tonsillectomy and tongue base mucosectomy (ipsilateral oropharyngeal MALTectomy) and" is removed.
- 3c and 3d Surgery or radiotherapy can both be considered primary treatments and each can be considered or recommended after primary treatment based on imaging or pathology reports. If radiotherapy is chosen as primary modality neck dissection should not occur before.
- Agree with 'consider' but MDT not convinced of evidence for p16 negative disease- this remains a case by case discussion
- This may be better addressed in a consensus meeting about 'management of the neck', regardless of CUP or identifiable primary site.
- 3ai there was no majority answer it was split 50/50 agree/disagree

Round 2

- Use ultra sound scan to follow up contolateral neck
- Contra lateral staging neck dissection adds morbidity and unnecessary for well-lateralised tonsil SCC unless there are guarantees oncology will omit contra lateral RT.
- TBM in HPV negative tumours has low diagnostic yield so will advocate mainly for HPV positive unknown cancers.
- 3aii: agree only as "consider" used.
- There was a suggestion to core neck lumps before FNA. I would not agree, provided FNAs reported quickly. Our pathologists will FNA in clinic and then we will get an immediate result and plan accordingly.
- Whilst we would not get a PET-CT as a first investigation, we have a low threshold to early diagnostic PETs and have found them very helpful (eg "branchial cyst" in right age group will get PET after USS and FNA (both done in the neck lump clinic). We have easy access to PET with a short turnaround. If other centres do not have this facility then surely this is something we should recommend so the resources are made available to all that need it.
- I remember from the consensus day a few year years ago we agreed bilat tonsillectomy for all HNSCCUP, (in the days before HPV). Currently we certainly would if P16+ on core. In most instances we remove contralateral tonsil in P16+ patients even when primary identified.
- 3e could read "Consider contralateral staging neck dissection - following MDT discussion - where there is no clinical or radiological evidence of disease - for patients in whom a pN0 contralateral neck dissection would permit the omission of prophylactic dose radiotherapy"
- 3aii - why do we need this in here? There are so many potential "considers" for possible surgical interventions - where do we stop? This looks like more of a justification for considering this intervention for this patient group. I think the only contentious element to this statement is the role of BoT mucosectomy in the HPV negative neck node scenario - that gets crowded out by the management of the neck question in the same statement.
- 3aii agree that small primary with small neck disease (T1N1) can be treated with surgery alone. 3e excessive morbidity
- MDT just wanted to reiterate that all patients should be offered access to high quality clinical trials that will address the current lack of evidence to guide future decision-making.
- I think this depends on the primary stage and site
- Our MDT was divided on 3aii, but consensus decision is as above.
- Same comments as mentioned in the previous round
- Diagnosis and treatment should be separate
- Is HPV status on p16 or PCR? Will influence decision making if p16 only is used. Why only ipsilateral tonsillectomy for 3aii?

Round 3

- 3e I would think most centres would offer DXT rather than surgery but having consider is the correct way to leave this.
- 3aii agree - minimal morbidity and offers a reasonable chance of cure
- 3e disagree - unnecessary surgical morbidity
- 3aii - I feel this should only be included if the plan is to treat with surgery alone

- For 3aii: Disagree on the basis of the demographic representation of this group. Usually older less fit patients. Seems to much to consider all this treatment as they would likely be best served with non surgical treatment, Very different to the P16+ group.
- For 3e: Disagree on basis would offer a lot of unnecessary morbidity if PET negative. PET is a good NPV test as long as the primary lesion is PET Avid
- 3aii: Not clear from the question, but this would be with the expectation of RT as well.
- Agree with 'consider' as this allows treatment to be individualised to patient.
- The problem with recommending ipsilateral tongue base mucosectomy for all patients is that this is a diagnostic procedure; while it will yield a higher rate of identification of the primary tumour it has not demonstrably improved oncological outcomes. Recommending an invasive diagnostic modality, with significant resource implications, in the absence of clear evidence of benefit is not responsible in a 3rd party payer healthcare system. The correct setting for this procedure is a trial designed and powered to determine whether or not it confers benefit w.r.t loco regional control, disease specific, and overall survival.
- In our MDT we would not offer CL RT to these patients - as long as well-lateralised.
- In an unknown primary HPV negative SCC with small volume nodal metastasis and no ECS, radical radiotherapy should be considered. If there is large volume disease in the neck then surgery and adjuvant treatment as such HPV negative disease do not do as well oncologically with only RT.
- Contra lateral staging neck dissections add extra morbidity and more evidence is required.
- Both statements are reasonable practices for MDTs to discuss. "Consider" would be appropriate and recognises that alternative management plans are also reasonable.
- Contralateral neck dissection represents an acceptable rationale to obviate the need for radiation therapy: particularly in the context of treating the patient either: 1. solely with surgery or 2. through obviating bilateral neck radiation, if ipsilateral radiation is required post treatment.
- 3aii - No – This should be worked up as unknown primary and therefore bilateral TBM +/-bialteral tonsillectomy before ND

4: Non-surgical management of patients diagnosed as HNSCCUP

Unless otherwise specified, the HNSCCUP patients referred to in this session are assumed to have undergone an adequate diagnostic work-up, as per their MDT, and are due to commence treatment as a true HNSCCUP.

No.	Statement	Indicative vote	Round 1	Round 2	Round 3	Outcome
4a	Consider omitting adjuvant radiotherapy after an ipsilateral neck dissection where there is a solitary involved node less than or equal to 3cm with no extranodal extension.	[90.2%][b]	[100%]	-	-	Strong agreement
4b	Offer adjuvant radiotherapy +/- chemotherapy to the ipsilateral neck after an ipsilateral neck dissection where there is one node greater than 3cm, or there is more than one node involved, or where there is extranodal extension	[95.1%][b]	[94.7%]	-	-	Strong agreement
4c	Consider adjuvant radiotherapy +/- chemotherapy to bilateral neck after an ipsilateral neck dissection where there is more than one node involved or where there is extranodal extension	[90.2%][b]	[85.7%][c]	-	-	Strong agreement
4d	Consider radiotherapy +/- chemotherapy to the bilateral neck if there are multiple involved ipsilateral nodes or there is radiologically obvious extranodal extension	[93.4%][b]	[87.5%][c]	-	-	Strong agreement
4e	Consider including the ipsilateral oropharynx in the treated volume when giving radiotherapy to the neck for unilateral HPV positive HNSCCUP	[83.9%][a]	[94.6%][c]	-	-	Strong agreement
4f	Consider including possible mucosal primary sites when giving radiotherapy to the neck for unilateral HPV negative HNSCCUP. Decide possible sites based on pattern of nodal involvement and other clinicopathological features (eg smoking)	[87.1%][a]	[89.3%][c]	-	-	Strong agreement
4g	Offer 50Gy in 2Gy fractions or equivalent* as the radiotherapy dose for possible mucosal primary sites when they are intentionally included in the target volume<br>*eg 54Gy in 30 fractions or 56Gy in 35 fractions	[95.1%][b]	[91.1%][c]	-	-	Strong agreement
4h	Offer concomitant cisplatin chemotherapy with primary radiotherapy if there are multiple involved nodes or obvious extranodal extension and the patient is suitable to receive cisplatin	[96.7%][b]	[96.4%][c]	-	-	Strong agreement
4i	Offer concomitant cisplatin chemotherapy with adjuvant radiotherapy if there is pathological extranodal extension and the patient is suitable to receive cisplatin	[96.7%][b]	[100%][c]	-	-	Strong agreement
4j	Include the ipsilateral retropharyngeal and retrostyloid nodes in the elective target volume when giving radiotherapy to the ipsilateral neck where level II is involved	[93.4%][b]	[94.5%][d]	-	-	Strong agreement

[a] n=62, [b] n=61, [c] n=56, [d] n=55

Comments on Section 4

- 4c to 4g need to be taken into context with what has been done surgically. If a full MALTectomy has be done and is negative, then these statements could be considered but may not be followed. What is suggested may be common practice. What about turning them the other way round and considering NOT treating the opposite neck etc if a full MALTectomy has been done?
- - 4b - this is too broad based - majority of patients will fall within this. It would be beneficial to stratify this group and offer different treatment option based on 1. >3 cm 2. Multiple nodes 3. Extracapsular extension. The clinical outcome is not same in all these three group of patients.
- 4h- consider? I think HPV+ N2b disease does not mandate CRT after surgery, but if primary treatment is RT I would agree offer chemo too.
- Not sure about 4i either. In HPV+ disease and "minor" ENE there may be no compelling reason for chemo in addition to RT. Perhaps should be a consider?
- 4j would depend whether there's any radiological evidence of RP nodal involvement, in which case I would recommend RT is given.
- Not sure where there is evidence to justify such a blank statement as this. Lots of papers out there with N2b disease treated ipsilateral showing a very low rate of primary and contralateral recurrence and for those who recur high rates of salvage. Think it weighing up the patient, toxicity, burden of disease and having informed discussion with patient. Don’t think you should be pushing oncologists routinely treat a patient with 2 or 3 nodes bilaterally with a statement like this There is only a point in increasing diagnostic scans and degree and complexity of diagnostic biopsies if it then changes what oncologists are willing to cover. With modern diagnostic techniques the certainty it is an unknown primary is high and therefore ipsilateral treatment without mucosal coverage and contralateral neck might be appropriate and justify the lengthy and morbid staging procedures and improved long term toxicity and functional outcomes without compromising survival. Xrt has always been based on 20% risk of microscopic disease and don’t think with modern staging the chance of primary or contralateral nodes is as high as that to continue justifying bilateral and mucosal xrt in unknown primary scc apart from very high risk (but could justify as they are at as much risk of distant mets or local failure than regional recurrence) or bilateral involved (rate) cases
- 4b - these 'inclusion criteria' for post op RT are quite wide - I'd suggest 'consider RT for 1 node >3cm' whereas the stronger 'offer RT' is more appropriate for the multiple LNs, ENE
- We do not routinely give RT to potential primary sites. Where it is given, we would stick to 50Gy equivalent - thise dose should be adequate for micropscopic disease. Note - for statement 33, updated RCR guidance being published soon
- Please note these responses incorporate the west of scotland regional mdts including NHS Greater Glasgow and Clyde, Ayreshire and Arran and NHS Lanarkshire
- 4a we would not omit RT if a primary has not be identified, we would omit RT to the neck in that scenario though
- 4(c). Radiotherapy to the contralateral neck (provided N0) can be avoided by performing a contralateral selective neck dissection under the same GA if the ipsilateral neck is being operated on prior to giving ipsilateral (C)RT.
- 4f - our MDT favoured TMI V no primary Site RXT considered on a case by case basis. Partial treatment may be incorrect and result in reduction of subsequent treatment options should a primary present.
- 4j - we would actually like to abstain from this as our 2 oncologists have respectfully differing opinions here
- 4b Offer rather than cosider if multiple nodes or extranodal extension. Consider if solitary node over 3cm with no extranodal extension.
- 4c and 4d We generally offer unilateral treatment unless there are multiple levels of nodes involved.
- 4c and 4d: although this is discussed and considered, the MDT wouldn’t recommend this unless there are other features to indifcate contra lateral treatment.
- 4g: some debate in the MDT. This is commonly out practise but some question as to evidence
- 4j: our practise is to include this when there is bulky level 2 disease. Feel ‘include’ is too strong with current wording.
- 4j needs more detail - is it p16+ disease, is it bulky nodal disease?
